# Potential problem and solution of lateral plate postposition for the posterolateral tibial plateau fracture

**DOI:** 10.1186/s13018-023-04397-x

**Published:** 2023-12-21

**Authors:** Zhenghui Hu, Weizhi Ren, Wen Zhang, Liubing Li, Wei Xu

**Affiliations:** 1 Department of Orthopedics, The Second Affiliated Hospital of Soochow University, No. 1055, Sanxiang Road, Suzhou, 215004 Jiangsu Provine China; 2 Orthopedic Institute of Soochow University, No. 333, Ganjiang East Road, Suzhou, 215004 Jiangsu Provine China

**Keywords:** Posterolateral tibial plateau fracture, Plate postposition, Outcomes, Finite element analysis

## Abstract

**Background:**

There has been controversial for the treatment of the posterolateral tibial plateau fractures (PTPF). This study aimed to evaluate clinic outcomes of the lateral locking compression plate (LCP) postposition, analyze the feasibility of LCP postposition through anatomical measurement, and address the potential problems of LCP postposition through the biomechanical assessment.

**Methods:**

39 patients with PTPF undergoing LCP fixation between June 2019 and June 2022 were retrospectively evaluated. All cases were divided into two group: Group A (15 cases) employed plate transverse arm postpositioning with posterolateral (PL) fracture fixation using two raft screws, while Group B (24 cases) utilized non-postpositioning with fixation by a single raft screw. Surgical duration, intraoperative blood loss, the change of lateral tibial plateau angle (LTPA), lateral tibial plateau posterior slope angle (LPSA) and fracture collapse between immediate postoperative and last follow up, range of motion (ROM), HSS knee score, and Lysholm knee score were recorded. CT measurements of the fibular head superior space and LCP transverse arm were taken in 50 healthy adult knees to assess postposition feasibility. Finally, three fracture models were established using finite element analysis: Model A with plate postposition and PL split fracture fixed by two raft screws of transverse arm, Model B with plate non-postposition and PL split fracture fixed by one raft screw, and Model C with plate non-postposition and PL split fracture fixed by one raft screw and anterior–posterior tension screws. Loadings of 250N, 500N, and 750N were applied for the analysis of the displacement degree, von Mises stress distribution.

**Results:**

Results indicate comparable operative duration and intraoperative hemorrhage between groups. Complications were minimal in both groups. Group A demonstrated superior outcomes in terms of radiographic parameters, functional scores, and fracture collapse prevention. CT measurements revealed compatibility in 72% of healthy knees with the postpositioning technique. Finite element analysis indicated favorable biomechanical stability.

**Conclusion:**

Not all patients with PTPF were applicable to the management of the plate postposition and two raft screws fixation, even though this technique exerted good biomechanical stability and achieved satisfactory clinic outcomes. When the PL fracture was fixed by only raft screw through LCP owing to various reasons, two anterior–posterior tension screws might be necessitated to maintain the fracture stability.

## Introduction

The posterolateral tibial plateau fractures (PTPF), either isolated or combined with another column fractures, is common than previous reports with the widespread use of computed tomography (CT) for identification. Some studies reported that PTPF accounts for 54.3% of lateral tibial plateau fractures involved the posterolateral (PL) column and about 15% of all tibial plateau fractures [1]. The management of displaced PTPF is a challenge to orthopedic surgeon. Due to common peroneal nerve, fibular collateral ligament (FCL), and the fibular head adjacent to fracture region [2–4], the visualization and reduction of PTPF is difficult and suboptimal fixation or unsatisfactory outcomes occur. How to address PTPF is intractable and is also a controversial topic [5].

Different approaches, alternative fixation techniques and implant devices are implemented for addressing PTPF [6–9]. The posteromedial, anterolateral, or PL approach often are adopted the treatment of PTPF [10–12]. Each surgical method has its own advantages and drawbacks. For example, the placement of the posterior supportive plate can provide the highest biomechanical stability for PTPF through posterolateral or posteromedial approach [5, 7, 13], but increase iatrogenic injury compared with the placement of lateral plate through anterolateral approach [14].

Anterolateral operative approach is relatively simple, with low risk of injury to the neighboring structures. However, it is still controversial to the lateral plate for the PTPF fixation owing to the weakness of the anti-shear effect. Hu et al. introduced a method involving the creation of a space in the superior aspect of the fibular head and positioning the lateral plate posteriorly through anterolateral approach, thereby increasing the number of the screw for fracture fixation and enlarging the purchased area of PL fracture fragments [15]. However, there are some potential problems that need to be addressed. Can the difference of height, sex, and anatomical structure affect the posterior placement of lateral plate for PTPF in clinic? How about the clinical outcome, when PTPF is fixed with the different number of raft screws due to fracture size, even the plate postposition? How to provide the better biomechanical stability when only one raft screw on plate purchase the main fracture fragment, even the plate postposition for PTPF. In this study, we collected the clinical data of the patients with PTPF treated with the posterior placement of lateral plate fixation in our hospital. We also conducted anatomical measurements in 50 intact knees without fracture by CT to analyze the feasibility of lateral plate postposition and performed finite element analysis of the different fixation for PTPF. These results could guide clinical practice and promote effective treatment for the fracture involved with PL tibial plateau.

## Materials and methods

This study was reviewed and approved by the Ethics Committee of the Second Affiliated Hospital of Soochow University completed on June 29, 2023 (Approval File No. JD-HG-2023-55). Informed consent was obtained from all participants in this study.

### Surgical procedure

**Fig. 1:**
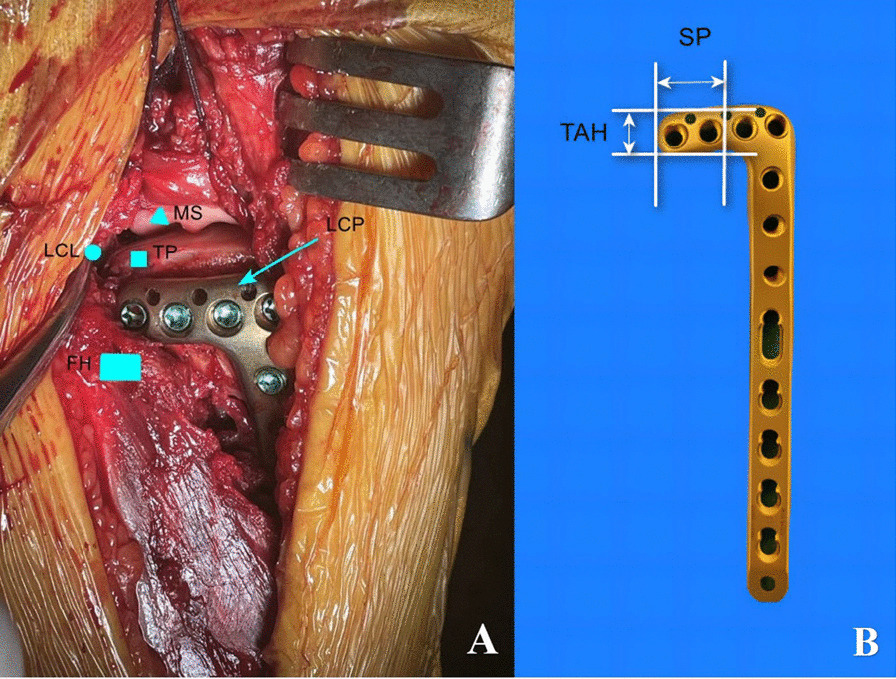
**A** Intraoperative visualization showed postposition plate for posterolateral tibial plateau fracture fixation via trans-supra-fibular head approach. TP, tibial plateau; FH, fibular head; LCL, lateral collateral ligament; MS, lateral meniscus; LCP, locking compression plate; **B** Appearance and measurement of LCP by DePuy Synthes. SP, distance from the penultimate screw hole of the transverse arm of the plate to the end of the transverse arm; TAH, the height of the transverse arm

The patient was positioned in a supine position. After sterilization and draping, the tourniquet was used. An anterolateral skin incision of knee was made, approximately 10 cm in length, crossing Gerdy's tubercle and extending posterosuperiorly to the fibular and joint line. The space between FCL and the lateral tibial plateau was developed. The inferior border of the coronary ligament and joint capsule were opened, and the lateral meniscus was proximally retracted using sutures. The FCL was mobilized to retract posteriorly with knee semi-flexed and the knee was in varus position, allowing exposure of the whole of the lateral tibial plateau structures **(**Fig. 1A**)**. The depressed articular fragment was elevated to restore the congruence of the tibial plateau through a created cortex window about 5 cm distal to the joint line and the K-wire was used to maintain the reduction. Alternative bone void fillers was used to fill metaphyseal defects and add stability to articular surface reduction. The reduction forceps was used to reduce the PL split fracture and the K-wire fixed it temporarily. A 3.5-mm L-shaped locking plate, the transverse arm of the plate with 4 holes, was placed as posteriorly as possible, through the superior fibular head space. The main PL fracture fragments were fixed with the locking raft screws. The operative wound was then rinsed thoroughly, a drainage tube was placed, and the incision was closed.

### Patient series

The inclusion criteria were as follows: All patients had isolated lateral tibial fractures (Schatazker I-III) involving PL quadrant and managed at our institution between July 2019 and June 2022, with preoperative and postoperative imaging, including knee X-ray, computed tomography (CT), and a minimum postoperative follow-up of 12 months. All operations of lateral locking plate postposition fixation for PTPF were performed. The exclusion criteria were as follows: patients had previous surgery on the affected knee, fractures involving the medial tibial plateau or the whole tibial plateau (Schatazker IV–VI), open fractures of tibial plateau, pathological fractures of tibial plateau, fractures of tibial plateau with neurovascular injury, and knee arthritis before injury. Finally, 39 patients were recruited, and complete follow-up results were obtained.

**Fig. 2:**
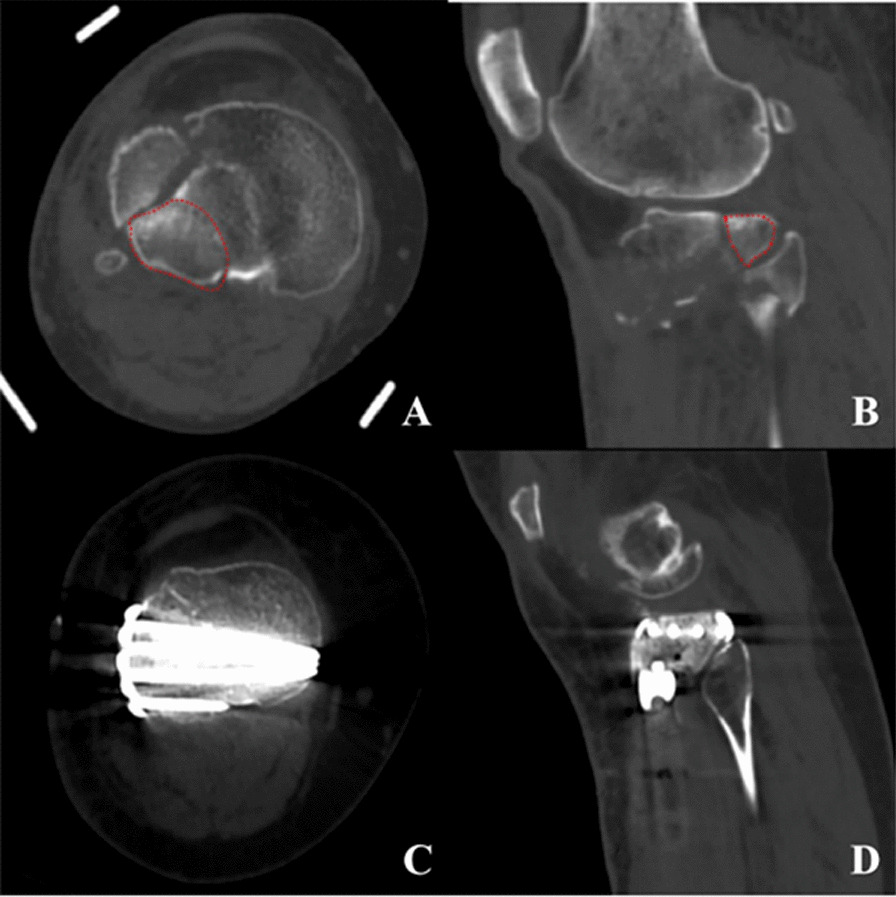
CT Image of Schatzker type II tibial plateau fracture involving the posterolateral plateau. **A** Preoperative transverse image, **B** preoperative sagittal image, **C** postoperative transverse view showing plate posterior placement up to two screw fixation, **D** postoperative sagittal view showing posterior placement of the plate up to two screws for fixation

**Fig. 3:**
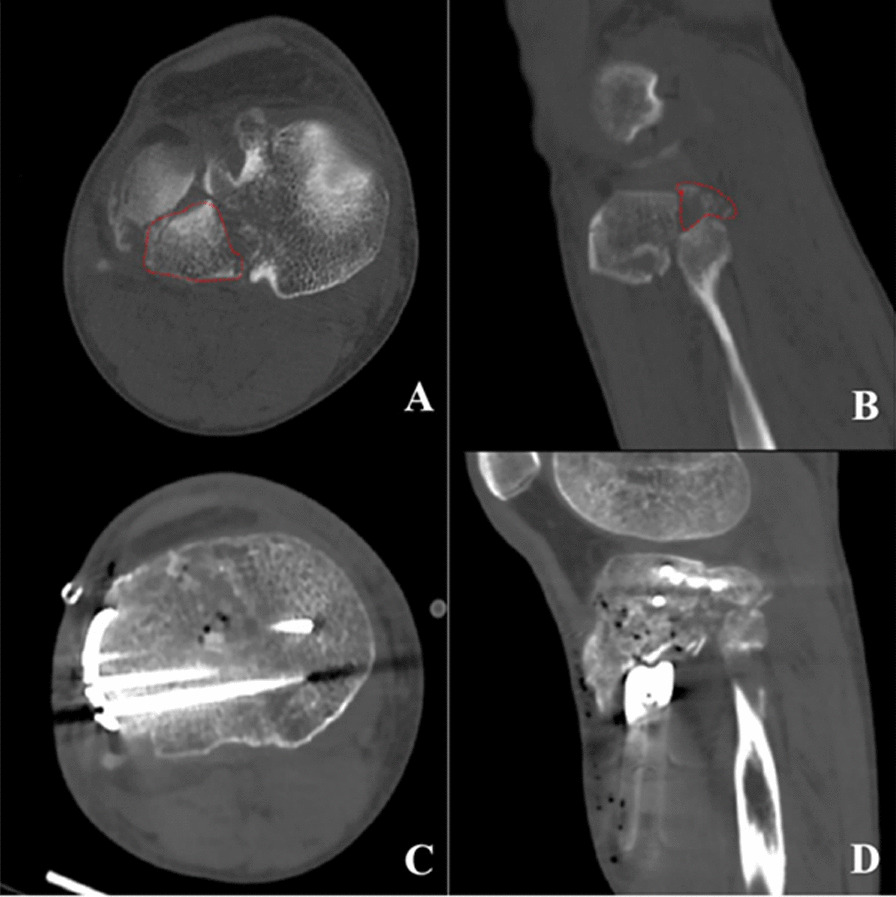
CT Image of Schatzker type II tibial plateau fracture involving the posterior lateral plateau. **A** Preoperative transverse image, **B** preoperative sagittal image, **C** postoperative transverse view showing the plate not posteriorly placed with only one screw fixation, **D** postoperative sagittal view showing the plate not posteriorly placed with only one screw fixation

**Fig. 4:**
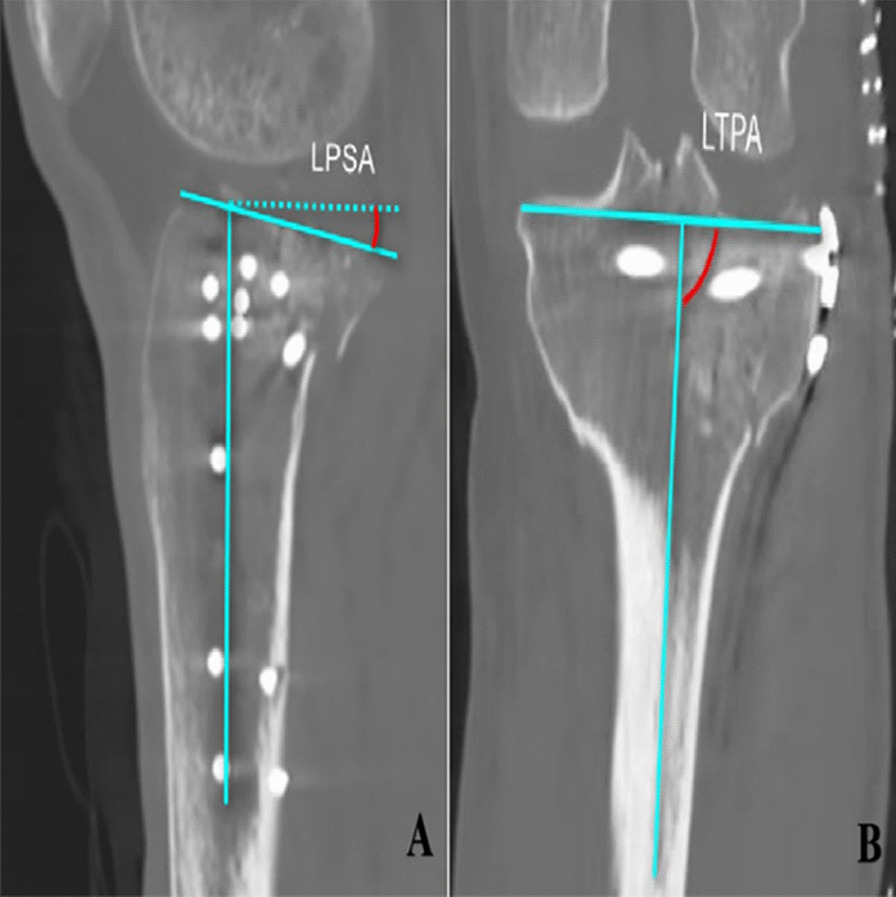
A schematic illustration depicting the procedure for measuring lateral tibial plateau posterior slope angle and the lateral tibial plateau angle is provided. **A** LPSA, stands for the angle formed between the line of the articular surface of the tibial plateau and the vertical line of the tibial shaft. **B** LTPA, defined as the lateral pinch angle formed between the axis of the tibia and the line connecting the medial and lateral plateau

39 patients were divided into two groups. One group was the patients whose main PL fracture fragment was grasped by two locking screws **(**Fig. 2A–D**)**. Another group was the patients whose main PL fracture fragment was grasped by only one locking screw **(**Fig. 3A–D**)**. The operative time, the volume of blood loss, preoperative and postoperative x-ray and computed tomography (CT) findings were recorded. The patients were followed up at 1, 3, 6, 12 months postoperatively, with clinical and radiographic evaluations of the fracture healing and complications. The Hospital for Special Surgery (HSS) knee score, the Lysholm knee score and the range of motion (ROM) were used to evaluate postoperative clinical outcome. Radiologically, Lateral tibial plateau angle (LTPA) and lateral tibial plateau posterior slope angle (LPSA) were compared between the immediate postoperative and last follow up **(**Fig. 4A, B**)**.

### Statistical analyses

The statistical analysis was conducted using SPSS 20.0 software. Normality of the measurement data was assessed using the Shapiro–Wilk test. The results were presented as mean ± standard deviation. Intra-group comparisons were analyzed using the paired *t* test, while inter-group comparisons were evaluated using the independent samples *t* test. Count data were expressed as percentages, and comparisons were made using Fisher's exact test.

### The measurement of the superior space of the fibular head in CT and the locking plate

We randomly enrolled a total of 50 patients (25 male: 25 female) undergoing the CT examination of knee in our institution between January 2022 and January 2023. The patients meeting any of the following criteria were excluded from our study: 1. history of previous knee surgery; 2. history of previous knee injury; 3. congenital knee abnormalities; and 4. severe and obvious knee disease such as osteoarthritis, rheumatoid arthritis, gouty arthritis. In CT imaging, a uniform scanning protocol was uniformly applied to all subjects. Subsequently, the DICOM formatted images were imported into RadiAntViewer software and reconstructed utilizing the transverse line of medial and lateral condyle as the reference baseline. The ensuing anatomical measurements were conducted in the reconstructed sagittal position. CT images from 50 patients were meticulously curated by two radiologists and an experienced surgeon. Before embarking on measurements, they underwent rigorous training to ensure adeptness and uniformity in measurement techniques.

**Fig. 5:**
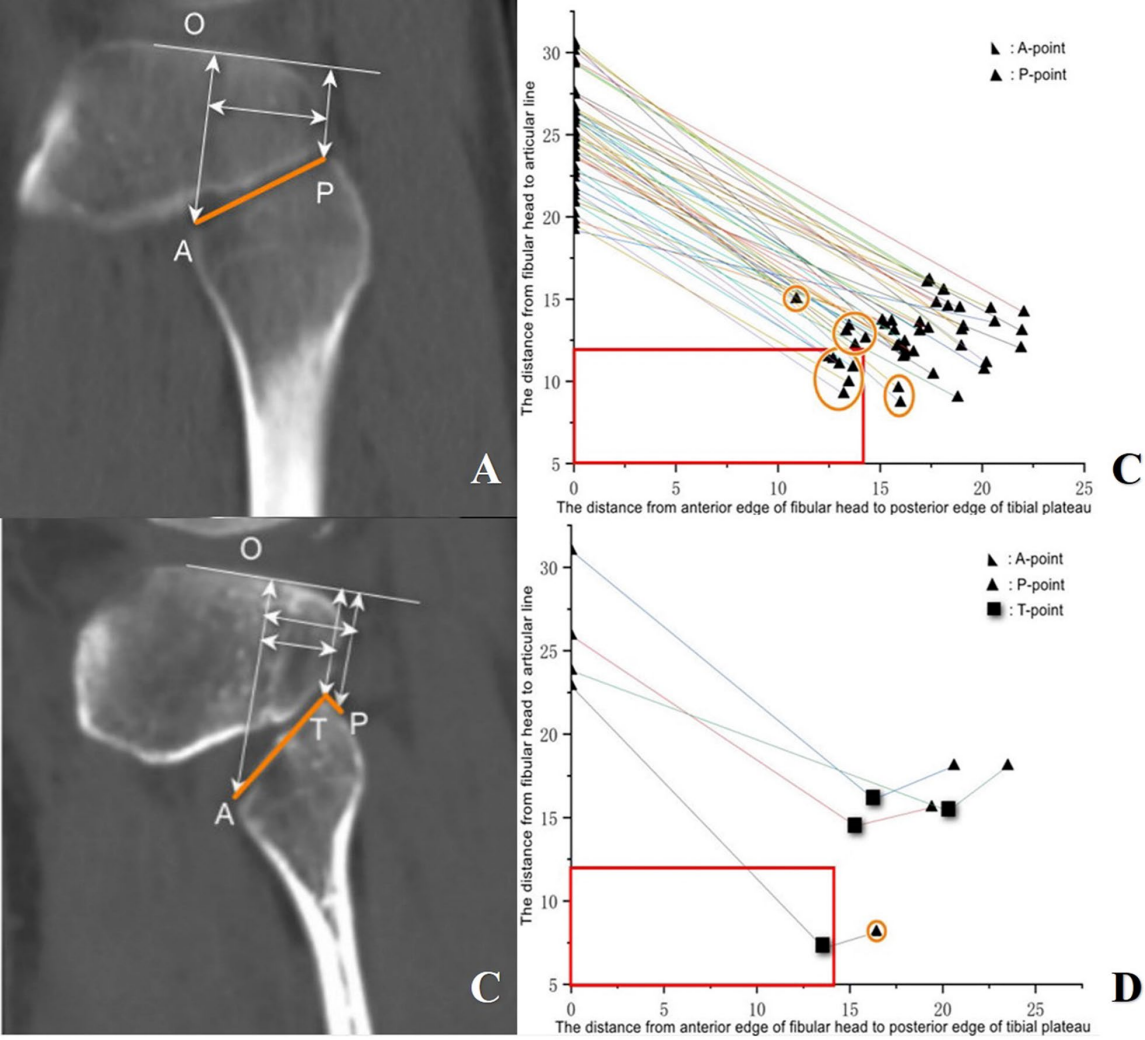
Morphometry and coordinate system analysis of CT-based 2D reconstruction of sagittal fibular head and posterior tibial plateau map. **A** Sagittal view illustrating the posterior position of the apex of the fibular head relative to the posterior border of the tibial plateau. **B** The posterior edge of the tibial plateau was positioned anteriorly to the apex of the fibular head in 46 intact knee. Coordinate system showing corresponding plate positions and the distance of AO and PO which represent superior space of the fibular head. **C** Sagittal view illustrating the anterior position of the top of the fibular head relative to the posterior border of the tibial plateau. **D** The posterior border of the tibial plateau was situated posteriorly to the apex of the fibular head in 4 intact knee. Coordinate system showing corresponding plate positions and the distance of AO, PO and TO which represent superior space of the fibular head. Point A, the foremost point of the anterior edge of the fibular head; Point P, the intersection of the plumb line of line O at the posterior edge of lateral tibial plateau with the fibular head; Point T, the apex of the fibular head; Line O, articular surface line; Orange Round, Cases of non-compliance

The fibular head was the mark of PL quadrant of tibial plateau. In the reconstructed sagittal view, O line was the articular surface line of lateral tibial plateau. The point of A was the highest point of the anterior edge of the fibular head. Point P is the intersection of the plumb line of line O at the posterior edge of lateral tibial plateau with the fibular head and Point T was the apex of the fibular head. The distance of AO, PO and TO from point A, P and T to line O and the distance of AP and AT from point P, T to Point A parallel to Line O were measured, respectively **(**Fig. 5A and C**)**. Initially, the fibular head morphology was examined, where the posterior edge of the tibial plateau was positioned anteriorly or posteriorly to the apex of the fibular head (denoted as the P-point located anteriorly or posteriorly to the T-point). Subsequently, two groups was divided according to the fibular head morphology. In one group, P-point anterior to T-point, the distance of PO determined whether there were enough space to insert the transverse arm of the plate posteriorly. In instances P-point posterior to T-point, the posterior border of the tibial plateau was situated posteriorly to the apex of the fibular head, the fibular head could block the insertion of the transverse arm of the plate if the distance of TO was less than that of PO and the distance of AT was less than that of AP **(**Fig. 5A and C**)**. We supposed that FCL could be retracted posteriorly under partial knee flexion, consequently comprehensive exposure of the entire lateral tibial plateau and lateral plate postposition was determined by the distance between articular surface and fibular head.

We proceeded to measure a commonly employed Locking Compression Plate (LCP) by DePuy Synthes (USA), a prevalent choice for lateral tibial plateau fractures. The height of the transverse arm (TAH) of the LCP and the distance between the penultimate screw of the transverse arm and the end of the transverse arm (SP) were meticulously measured utilizing a specialized vernier caliper **(**Fig. 1B**)**. We compared the TAH with AO, PO, and TO value. When the value of TAH was less than that of AO, PO, and TO, that meant the space in the superior aspect of the fibular head was large enough and the transverse arm could be placed posteriorly. Likewise, the distance of SP and AP was compared. When the distance of SP was less than that of AP, that meant two raft screws could fix the PL plateau. Conversely, if the distance of AP was less than that of SP, only one raft screws through transverse arm could fix the PL plateau, even the lateral plate postposition.

### Finite element analysis

#### Equipment and software

We selected a male 30-year-old healthy adult and scanned the knee joint by CT images. Subsequently, a tibia model was constructed using the threshold segmentation, region growth, and 3D reconstruction in Mimics20.0 software, a cancellous bone model in 3-matic12.0 software, and a solid model in Geomagic12.0 software. The model was imported into ProEngineer 5.0 software. Internal fixation models, lateral tibial locking compression plate, were created in the software. We imported all the models into Hypermesh 2017 software and divided all the parts into tetrahedral mesh Solid187, in which the unit size of the cortical bone and cancellous bone models were 1 mm, and the plates and screws were 0.5 mm, the material properties as described by Huang [16]. Three-dimensional modeling of plates and screws was performed according to the manufacturer's skeletal specifications using the computer-aided software Creo Parametric (PTC, Inc., United States). Any contact between the plate and the fracture fragments was defined as frictional contact. Following Viceconti et al. [17] we set the coefficient of friction between bones at 0.4. We imported the model into the software Geomagic Studio (3D System Inc., Rock Hill, SC, United States). Modeling Tibial Plateau Split Fractures by Simulating Fracture Lines, the fracture model and internal fixation model were based on the previous study proven to be effective [18].

#### Modeling of posterolateral split fracture of tibial plateau and setting the load level

**Fig. 6:**
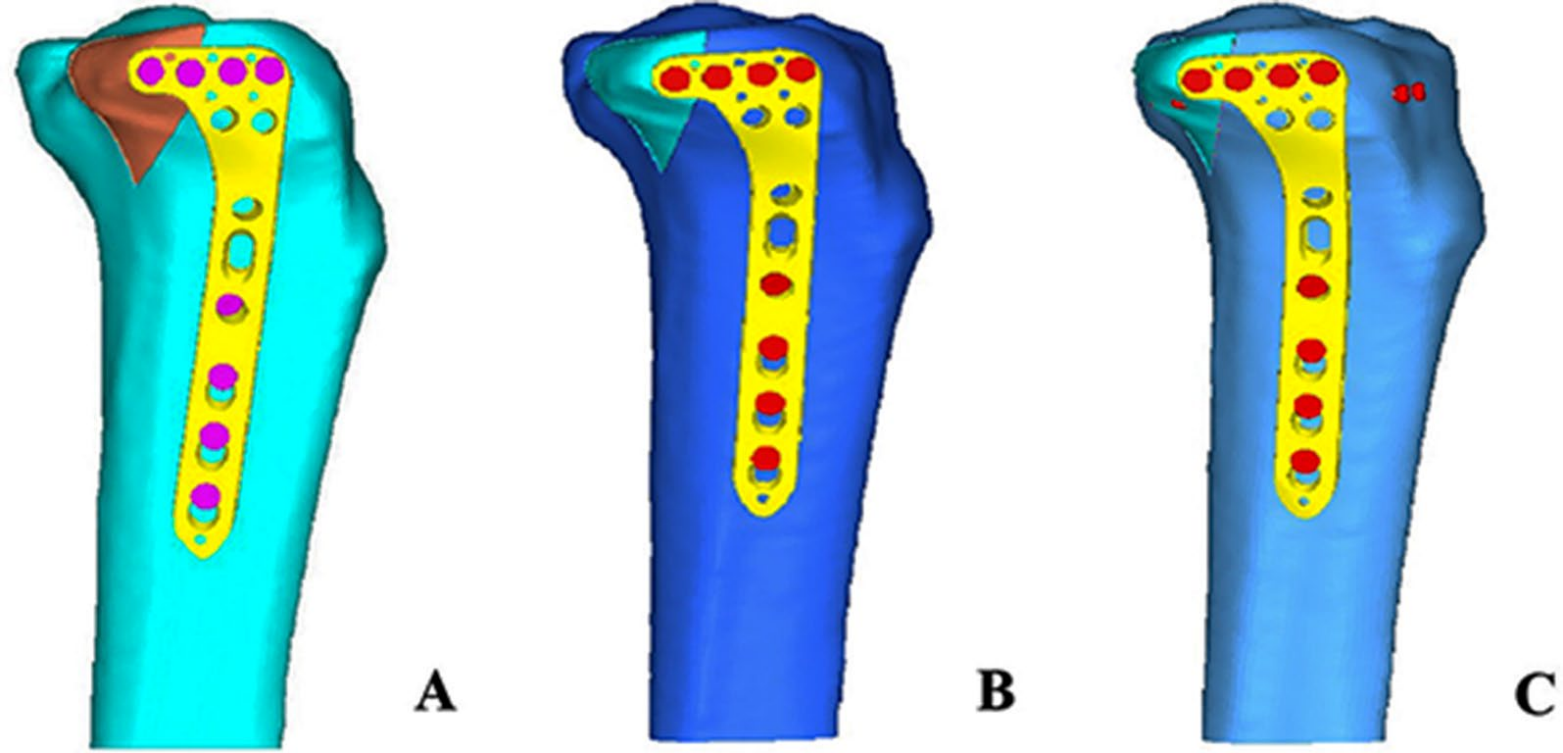
The pattern of three types of fixation for PL fractures. **A** LCP postposition and PL fracture fixed by two raft screws. **B** LCP placed non-postposition and PL fracture fixed by one raft screw fixation. **C** LCP placed non-postposition, PL fracture fixed by one raft screw and two anterior–posterior tension screws

Three groups of fracture models showed: Group A with posteriorly placed plates (split fracture fixed by two raft screws through the plate), Group B with the plate placed commonly (split fracture fixed by only one raft screws through the plate), and Group C with the plate placed commonly (split fracture fixed by only one raft screws through the plate) and supplemented by anterior–posterior tension screws **(**Fig. 6**)**. Compression of PL fractures by three different loads, the fracture models were analyzed and validated by the software ANSYS Mechanical APDL 19.0 (ANSYS, Inc., United States). For an adult weighing 60 kg, the typical biomechanical load on the knees is approximately twice their body weight. Additionally, in an upright walking gait, the medial platform takes up about 55%, while the lateral platform comprises approximately 45%. In this context, each leg is expected to support twice the body weight during normal adult walking [19, 20]. Thus, when 1–3 times the body weight of its lateral platform load is about 250N, 500N, and 750N, we choose three different load levels; 250N, 500N, and 750N to simulate the pressure load of the posterior lateral platform.

## Results

### The patients with PTPF fixed by two raft screws had better radiological and clinic outcome

There were no discernible differences in operative duration and intraoperative hemorrhage and follow-up time between two groups of patients with PL tibial plateau fracture (*P* > 0.05). Encouragingly, complications such as vascular nerve injury, internal fixation failure, fracture nonunion or malunion, and lower extremity deep vein thrombosis were absent in both cohorts. At last follow up, the incidence of collapse exceeding 2 mm or inadequate repositioning was 0 (0%) in the group with two screws fixation and 7 (29.167%) in the group with one screw fixation, exhibiting a statistically significant (*P* < 0.05). In the group with two screws fixation, the assessments of immediate postoperation and last follow-up revealed non-significant variations in LTPA and LPSA (*P* > 0.05). Conversely, in the group with one screw fixation, a statistically significant difference in both LTPA and LPSA between immediate postoperation and last follow-yp was observed (*P* < 0.05). When comparing between two groups, a notable distinction in LTPA and LPSA was identified (*P* < 0.05). Furthermore, statistically significant disparities were evident in Range of Motion (ROM), HSS knee score, and Lysholm knee score between two groups (*P* < 0.05) and the group with two screws fixation had better clinic outcomes than the group with one screw fixation (Table 1).

**Table 1:** Radiologic and clinical findings in both groups

	PTPF with two screws fixation (*n* = 15)	PTPF with one screws fixation (*n* = 24)	*P* value
Follow-up time (months)			
Age (years)	51.8 ± 13.79	54.40 ± 11.95	0.538
Gender, Male/Female	5/10	10/14	0.570
BMI (Kg/m^2^)	24.88 ± 2.408	25.17 ± 2.385	0.704
Surgical time (min)	83.0 ± 14.96	90.32 ± 14.19	0.123
Intraoperative blood loss (ml)	88.7 ± 20.37	102.0 ± 31.26	0.142
Postoperative collapse (> 2 mm)	0/15	7/17	0.031*
LTPA (°) (Postoperative)	89.56 ± 2.128	86.32 ± 1.796	
LTPA (°) (last follow-up)	88.38 ± 2.754	83.68 ± 1.725	
LTPA (°) (Difference)	− 1.188 ± 2.971	− 2.640 ± 1.551	0.047*
*P* value	0.1308	< 0.0001*	
LPSA (°) (Postoperative)	9.188 ± 3.600	10.60 ± 3.797	
LPSA (°) (last follow-up)	9.313 ± 3.497	13.76 ± 3.940	
LPSA (°) (Difference)	0.1250 ± 1.408	3.160 ± 2.853	0.0003*
*P* value	0.7275	< 0.0001*	
ROM (°)	130.1 ± 14.31	118.0 ± 10.68	0.044*
HSS knee score	94.50 ± 3.633	90.04 ± 4.257	0.022*
Lysholm knee score	93.63 ± 5.818	88.96 ± 7.541	0.041*

### Plate postposition and two screws fixation could be implemented in about 70% cases with PTPF by CT measurement

The transverse arm of all LCP plate (DePuy Synthes) was uniform. TAH of the LCP was 12 mm and SP was 14 mm **(**Fig. 1B**)**. In CT measurement, we found the posterior border of the tibial plateau was situated anteriorly to the apex of the fibular head in most cases, PO represented the minimum value of the superior space of fibular head. If PO value was less than TAH (12mm), the LCP could not be completely placed posteriorly. We also found four cases that the apex of the fibular head was situated anteriorly to the posterior border of the tibial plateau. If TO value was less than 12 mm, the fibular head could block the postposition of the LCP **(**Fig. 5A–D**)**. On the other hand, if the distance of AP was less than SP (14 mm), the PL fracture could not be fixed by two raft screws through the LCP.

In Fig. 5B and D, the "red box" was marked with horizontal coordinates of 14 mm and vertical coordinates of 12 mm, which meant SP and TAH, respectively. Totally, 36 cases of 50 patients could meet both conditions, the plate postposition and two screws fixation.

### Two raft screws through the plate provided better stability than one raft screws, one raft screw through the plate and two tension screws compensated the weakness of stability

**Fig. 7:**
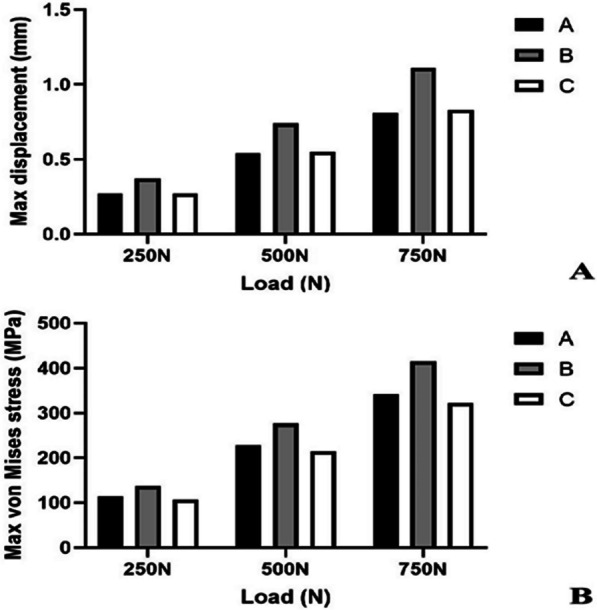
Deformation degree and von Mises stress distribution analysis in three fracture models under 250N, 500N, and 750N load conditions. **A** Deformations of three fracture models under various loads. **B** Max von Mises stress of three fracture models under various loads

**Fig. 8:**
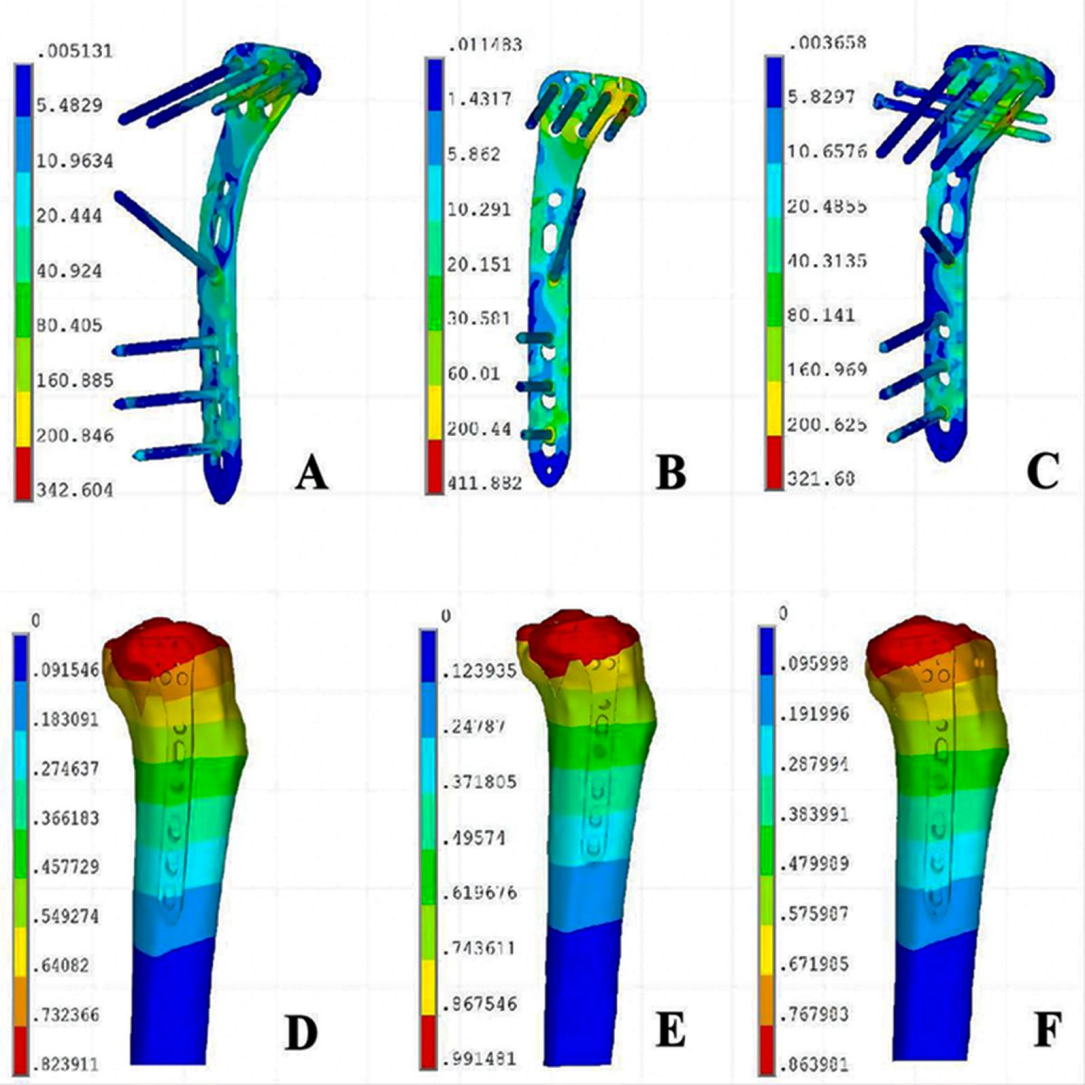
Stress distribution diagram and displacement field of the three finite element models. **A** Stress distribution of model A in posterolateral (PL) fracture. **B** Stress distribution of model B in posterolateral (PL) fracture. **C** Stress distribution of model C in posterolateral (PL) fracture. **D** Displacement field of model A in posterolateral (PL) fracture. **E** Displacement field of model B in posterolateral (PL) fracture. **F** Displacement field of model C in posterolateral (PL) fracture

The finite element analysis performed on the three fracture models demonstrated that the deformation of the internal fixation device escalated proportionally with the applied load. At the load level of 250N, 500N and 750N, the degree of displacement in group C was similar to that of group A and was better than that of group B (Fig. 7A). In terms of von Mises stress, group C was close to group A and was lower than group B at the load level of 250N, 500N and 750N (Fig. 7B). The group A and the group C could have more biomechanical stability than group B. The analysis demonstrates that in Group A, stress concentration primarily occurs at the last two screws and the junction between the transverse arm and longitudinal arm. Two screws distributed the stresses more effectively, leading to a more even stress dispersion (Fig. 8A). In Group B, stress is predominantly concentrated on the final screw and the junction between the transverse arm and longitudinal arm, resulting in localized high stress concentrations (Fig. 8B). In Group C, the supplemented tension screws were positioned nearly perpendicular to the shear plane of the PL fracture, facilitating the absorption of the majority of stresses in the transverse arm of the plate and thus achieving the requisite biomechanical strength. (Fig. 8C).

## Discussion

In our study, the patients with PTPF fixed by two raft screws through plate postposition had better clinic outcome. However, anatomical measurement showed there were about 70% cases to meet this condition, the plate postposition and two screws fixation. If there was only one raft screw to fix the PL fracture fragment, supplementary two tension screws could increase fracture stability.

PTPF is a common fracture type in clinic [4, 7]. Sun reported that about 75% tibial plateau fractures were involved with posterolateral quadrant [21]. Addressing PTPF was still a challenge and a controversial topic [5]. Full exposure, rigid fixation, less iatrogenic injury and reducing complications were hard to be achieved simultaneously. Some studies showed that posterior plates provided superior biomechanical stability for PTPF [4, 13]. However, this approach increased the risk of posterior nerves and blood vessels injury during surgery [22, 23]. The Frosch incision, posterolateral approaches, necessitated a broad incision, resulting in significant soft tissue disruption, prolonged recovery, and an increased potential risk for common peroneal nerve injury [1, 24, 25]. Additionally, certain osteotomy techniques, such as fibular osteotomy and femoral epicondylar osteotomy showed the effectiveness in enhancing the visibility of PTPF [26, 27]. Nevertheless, the osteotomy-based approach unquestionably elevated the procedural complexity and the potential complications, such as osteotomy unhealing [28].

Employed routinely in the management of lateral plateau fractures, the lateral approach was preferred by many orthopedic surgeons due to its simplicity, reduced trauma, and lower risk of iatrogenic injury [29, 30]. While LCP fixation for PTPF through lateral approach had less biomechanical stability compared with posterior supportive plate fixation. We redesigned a new locking plate, which modified LCP plate, adding a hook at the end of transverse arm, and showed an approximate biomechanical property of posterior supportive plate. Likewise, to improving the stability of LCP for PTPF, Hu recommended the plate postposition through the superior space of the fibular head so that this way could provide more raft screws through the plate to fix PL fracture fragment [15].

However, we couldn’t place the plate posteriorly sometimes due to the hindrance of fibular head and FCL. And we had to provide only one screw fixation through the plate for PTPF due to the fracture size or the individual difference now and then, even if the plate postposition. We studied retrospectively 39 patients with PTPF in our hospital and divided these patients into two cohorts. A group with 15 patients, wherein the plate was positioned posteriorly with up to two screws for fixing the PL fracture fragment. B group with 24 patients, where the plate was non-postposition, utilizing only one screw for PL fracture fragment fixation. The results showed that two-screw fixation for PTPF had better clinic outcome and resisted the fracture collapse compared with one-screw fixation. The PL fracture fragment fixed by two screws may lead to loss of fracture substance and affect the integrity of bone fragment. Does fixation of the PL fracture fragment by two screws affect fracture nonunion? In our study, we observed that 15 patients with a posterior lateral fracture fragment underwent fixation with two screws, and we did not encounter any cases of nonunion. Similarly, Chen et al. [31] employed a two-screw fixation approach for the posterior lateral fracture fragment using an extended anterolateral approach, and all patients achieved both bone union and osseous integrity. Likewise, Hu et al. [15] employed a two-screw fixation approach through the fibular head approach, and no cases of nonunion were observed. Conversely, within our cohort of 24 patients treated with a single-screw fixation, seven individuals experienced re-collapse during the final follow-up. This leads us to posit that single-screw fixation may indeed influence bone healing, attributable to the compromised stability resulting from inadequate fixation strength. Although plate postposition and two-screw fixation was proved to be effective for PTPF, there were only 38.5% (15/39) cases to meet these condition and to achieve better outcomes. In another word, more than 60% cases couldn’t obtain satisfactory outcomes through supra-fibular-head approach due to surgical technique, fracture size, the anatomy difference, height, sex, or other limitation.

Furthermore, removing the influence of surgical technique, fracture size and soft tissue condition, we evaluated the feasibility of plate postposition and two-screw fixation from the perspective of anatomy. Through the measurement of superior space of fibular head in the CT of 50 intact knee, our findings revealed that 72% cases could accommodate plate placement over the fibular head, enabling secure fixation via the two screws of the transverse arm across the PL tibial plateau. Approximate 30% cases couldn’t achieve the rigid fixation for PTPF just because of the difference of individual anatomy [32]. Therefore, we should attach importance to the potential problem of the plate postposition for PTPF.

What should we do, when only one raft screw couldn’t provide effective support to PL fracture fragment, even if the plate was posteriorly placed? Since PL fractures primarily result from a mechanism involving shearing due to flexion valgus axial compression forces [33], we classified the fracture line as a coronal shear fracture. We constructed three distinct sets of finite element models. One-screw fixation exhibited the least favorable biomechanical stability and localized high stress concentrations, which might explain observed fracture collapse and poor clinic outcomes. In contrast, two screws distributed the stresses more effectively, leading to a more even stress dispersion. The supplemented anterior–posterior tension screws shared the shear stress on the PL fracture with the last screw in the plate and thus achieved the requisite biomechanical strength. So the comparative analysis between Groups A and C showed that the displacements in both groups were in close proximity, and the von Mises stress values exhibited similar trends. Recently, Gao reported that Lateral locking plate plus anterior–posterior lag screws for PTPF had a good preliminary clinical results [30]. When the PL fracture was fixed by only one raft screw through LCP owing to various reasons, two anterior–posterior tension screws might be necessitated to maintain the fracture stability. In term of fracture fragment difference, two optional fixation can extend clinical applications. It must be acknowledged that plate postposition with the two-screw fixation is relatively easy to operate and the introduction of anterior and posterior tension screws unquestionably extends both the duration and complexity of the surgical procedure.

Admittedly, given the diverse array of fracture patterns, anatomical discrepancies, or other individual factors encounter in clinical practice, it may not be easy to utilize these two fixations for PTPF. Hence, it is advised that the surgeon conduct a preoperative assessment of the fracture fragment's dimensions via CT or MRI imaging. In situations with smaller fracture masses, two optional fixation may not purchase the PL fracture mass. Other suitable fixation should be considered.

## Conclusion

Not all patients with PTPF were applicable to the management of the plate postposition and two raft screws fixation, which exerted good biomechanical stability and achieved satisfactory clinic outcomes. One raft screw in the plate transverse arm combined with two anterior–posterior tension screws could compensate for the lack of fixation effect.

### Limitations

This study had the following limitations: Firstly, there were a limited number of patients with PTPF. More samples was needed to further evaluate the fixation solutions for clinical application. Secondly, our finite element analysis was based on previous anatomical measurements of the fracture line and was not representative of all fracture types. Thirdly, fracture morphology and fracture size, which was an important consideration for the surgical treatment of this type of fracture, was missed here. Future studies will conduct biomechanical tests through constructing various fracture models to assess biomechanical stability.

## Data Availability

The datasets used and/or analyzed during the current study are available from the corresponding author upon reasonable request.
